# 

**Published:** 1890-05-17

**Authors:** 


### SATURDAY, MAY 17th, 1890.

**The Qualification Question** - 87

**Words of Consolation.**—Helps to Gain Patience - 88

**A Provident Out-Patient Department.** By the Right Hon. the Earl of Derby, K.G. - 89

**The History and Capabilities of Herbal Simples.** By W. T. Fernie, M.D. IX.—Borage - 90

**Editor's Letter Box.**—Plausibility or Honesty? - 90

**The Doctor's Pulpit.**—Country v. Town Practice - 91

**Jottings from Sweden and Denmark** - 92

**Everybody's Page** - 93

**Notes and News** - 94

**Annotations.**—A Parson on Doctors.—May and Missionaries.—"Wake Up, Mary." - 96

**The Practitioner's Mirror and Retrospect:**

Medicine—Hepatic Fever - 97

Surgery—Surgical Treatment of Diphtheria - 97

Therapeutics—Medicinal Soap - 99

New Drugs, Appliances, and Things Medical—"Vinolia" Toilet Soap - 99

**Aix-les-Bains** - 99

**Passing Topics.**—Friend and Foe - 100

**Scraps and Gleanings** - 100

### EXTRA SUPPLEMENT—THE NURSING MIRROR.

**En Passant.**—Nursing at Kemerton—Sister Rose Gertrude—Institution Life—Short Items—Training in Asylums—Infirmary Nurses - xxv
**Nursing Lectures.** XII.—Conclusion - xxvi
**Appointments** - xxvi
**The Charge of Private Lunatics** - xxvii
**Examination Questions.**—Prize Answer for April - xxvii
**Everybody's Opinion.** — Uniforms for June — Asylum Nurses—Male Nurses—The Brassey Holiday Home - xxviii
**Notes and Queries** - xxviii
**Vacancies—Presentations** - xxviii
**Amusements and Relaxation** - xxviii

